# A Spinal Epidural Arteriovenous Fistula Treated With Onyx Transarterial and Transvenous Embolization Using the Hemiazygos Vein: A Case Report

**DOI:** 10.7759/cureus.102795

**Published:** 2026-02-01

**Authors:** Akina Hirohashi, Shunsaku Goto, Takashi Izumi, Masahiro Nishihori, Issei Takeuchi, Shinsuke Muraoka, Hiroki Shimuzu, Satoshi Ito, Nobuhiko Mizutani, Ryuta Saito

**Affiliations:** 1 Neurosurgery, Konan Kosei Hospital, Konan, JPN; 2 Neurosurgery, Nagoya University Graduate School of Medicine, Nagoya, JPN

**Keywords:** endovascular approach, onyx, spinal epidural arteriovenous fistula, transarterial embolization, transvenous embolization

## Abstract

A spinal epidural arteriovenous fistula (SEDAVF) often causes dilatation of the epidural venous plexus extending across 1-2 vertebral segments, frequently exhibiting intramedullary venous reflux. Herein, we report a case of SEDAVF characterized by multiple discontinuous dilated epidural venous plexuses extending across seven vertebral segments, accompanied by intradural venous reflux. A 53-year-old woman presented with gait disturbance. Spinal magnetic resonance imaging (MRI) and computed tomography angiography (CTA) revealed spinal cord edema, flow voids, multiple dilated epidural venous plexuses, and intradural venous reflux, leading to a diagnosis of SEDAVF. Treatment was performed based on the assumption that an L2 radiculomedullary vein (RMedV) with intradural reflux was responsible for the symptoms. Accordingly, Onyx transarterial embolization (TAE) was initially administered, but as the patient’s symptoms persisted, we performed another intervention, combining Onyx transvenous embolization (TVE) via the azygos and hemiazygos vein with Onyx TAE, which removed reflux into the RMedV and improved symptoms. Thus, in cases of SEDAVF with multiple dilated venous plexuses extending across several vertebral levels, identifying the responsible lesion and fully understanding the vascular anatomy is needed to ensure appropriate treatment. An endovascular treatment strategy focused on eliminating intravertebral reflux may lead to symptomatic improvement. As such, meticulous preoperative imaging interpretation and accurate identification of the symptom-causing lesions are crucial.

## Introduction

Spinal epidural arteriovenous fistula (SEDAVF) is a rare vascular disorder accounting for approximately 10%-20% of all spinal arteriovenous shunt lesions [1]. SEDAVFs form arteriovenous shunts within the epidural veins or the internal vertebral venous plexus, leading to venous congestive myelopathy due to intradural venous reflux, as well as compressive myelopathy or radiculopathy caused by enlarged shunted pouches and dilated epidural venous plexuses [2,3]. SEDAVFs have been reported in association with trauma, surgery, spinal canal stenosis, and disc herniation, with the arteriovenous shunt typically forming at the same vertebral level as the underlying condition [2,4]. Treatment strategies are generally classified into endovascular embolization via transarterial and/or transvenous approaches or surgical disconnection of the shunt; however, after initial diagnosis with MRI and CTA, a detailed understanding of the angioarchitecture, including feeders and drainers, based on spinal angiography, is essential for appropriate treatment planning [5-7]. In most cases, the epidural venous plexus is dilated over a limited extent, usually involving one to two vertebral segments [8]. In contrast, only a few reports have described SEDAVFs with extensive longitudinal distribution spanning multiple vertebral levels, particularly those characterized by several discontinuous epidural venous pouches accompanied by intradural venous reflux. Herein, we report an exceptionally extensive SEDAVF in which the arteriovenous shunt spanned seven vertebral levels and formed four discontinuous epidural lesions. While initial Onyx transarterial embolization (TAE) was insufficient to achieve clinical improvement, additional Onyx transvenous embolization (TVE) via the azygos and hemiazygos venous systems successfully reduced the shunt flow and resulted in a favorable neurological outcome. This case highlights the diagnostic and therapeutic challenges of extensively distributed, multifocal SEDAVFs and underscores the clinical value of combined transarterial and azygos/hemiazygos-based transvenous approaches in selected complex cases.

## Case presentation

In October 2023, a 53-year-old woman presented with bilateral lower leg pain, predominantly on the left side, that had persisted for 8 days. The pain was accompanied by progressive muscle weakness, which gradually worsened and led to gait disturbance, prompting hospital admission. On neurological examination at presentation, muscle strength in the lower legs was markedly decreased, with manual muscle testing (MMT) graded as approximately 1/5 bilaterally. Sensory examination revealed diminished pain and temperature sensation in both lower extremities. Deep tendon reflexes, including the patellar and Achilles tendon reflexes, were absent bilaterally. No bladder or bowel dysfunction was observed.

**Figure 1:**
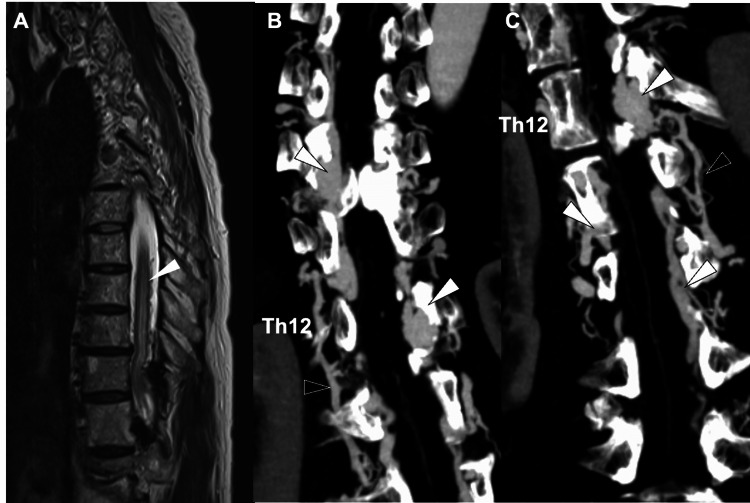
Pretreatment magnetic resonance imaging (MRI) and computed tomography angiography (CTA) findings A) Sagittal T2-weighted MRI demonstrating extensive spinal cord edema (arrowhead) and flow voids corresponding to dilated radiculomedullary vein (RMedV) on the dorsal aspect of the spinal cord. B) Coronal CTA images show dilated epidural venous plexuses along the right side of the spinal cord at the Th9–Th11 and L1–L2 levels (white arrowheads), appearing to be connected via the intervertebral veins and the azygos vein (black arrowheads). C) Similar findings are observed on the left side at the Th12 and L1–L3 levels, with connection through the intervertebral veins and the hemiazygos vein (black arrowheads).

Spinal MRI with T2-weighted sequences demonstrated flow voids and intramedullary high-signal intensity areas (Figure 1A). Computed tomography angiography (CTA) revealed four markedly dilated venous pouches extending over seven vertebral levels, with prominent spinal veins visible during the arterial phase, findings consistent with SEDAVF (Figure 1B, 1C). No cutaneous lesions corresponding to the vertebral level of the shunt were identified. Given the rapid clinical deterioration culminating in complete paraplegia of both lower limbs, urgent intervention was required. The neurological deficits were attributed to intradural venous reflux.

Because the lesion spanned multiple vertebral segments with complex inflow from numerous shunt points, an endovascular approach was considered more appropriate than direct surgery, as it allows both diagnostic evaluation and therapeutic shunt reduction in a single session. CTA further demonstrated that shunt flow within the dilated epidural venous plexus had induced thrombosis and subsequent occlusion of a portion of the internal vertebral venous plexus, with venous drainage rerouted through the hemiazygos vein (Figure 1B, 1C). In view of this complex venous anatomy, which was expected to make a transvenous approach technically challenging, an initial treatment strategy centered on TAE was planned.

**Figure 2:**
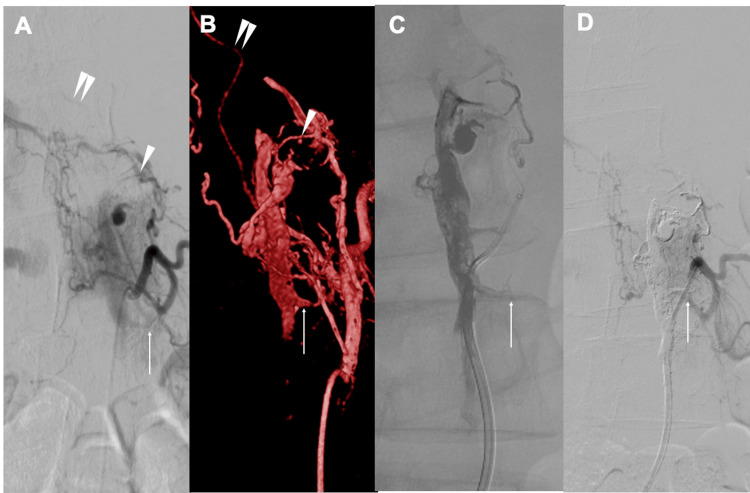
DSA observations and volume rendering technique image from 3DRA during the initial treatment. A, B) Selective angiography of the left L2 segmental artery demonstrated the retrocorporeal artery (white arrowhead) shunting into the epidural venous plexus (white arrow). The shunted venous plexus coursed inferiorly and drained into the RMedV (double arrowhead), exhibiting retrograde reflux. C) TAE was performed using Onyx18, and the injected material was confirmed to reach just proximal to the origin of the RMedV (white arrow). D) DSA after TAE demonstrating RMedV disappearance on a selective angiography of the left L2 segmental artery. RMedV: Radiculomedullary vein; DSA: Digital subtraction angiography; TAE: Transcatheter arterial embolization; 3DRA: 3D rotational angiography

A 6-Fr short sheath was inserted via right femoral artery puncture. Using a 4-Fr NUC1 catheter (Merit Medical Systems, Inc., South Jordan, UT, USA), bilateral segmental arteries from Th8 to L3 were angiographically evaluated. Thirteen segmental arteries were identified as contributors to the shunts, and dilated venous pouches segmented into four distinct regions spanning seven vertebral levels were visualized, consistent with SEDAVF. Selective angiography of the left L2 segmental artery demonstrated a retrocorporeal artery shunting into the segmented left internal vertebral venous plexus venous pouch extending from L1 to L3, with reflux into the L2 radiculomedullary vein (RMedV), resulting in venous outflow obstruction (Figure 2A, 2B). Given the direct reflux into the RMedV and the concordance with neurological deterioration, this site was considered the primary contributor to the patient’s symptoms. Angiography of the left Th12 segmental artery demonstrated an anterior spinal artery (ASA); however, the ASA was not visualized from other arteries. Therefore, approaches avoiding this artery were considered to carry a low risk of spinal cord infarction, and TAE was deemed feasible.

A 6-Fr ENVOY guiding catheter (Codman Neuro, Raynham, MA, USA) was positioned at the origin of the L2 segmental artery. A 4-Fr Cerulean G catheter (Medikit Co., Ltd., Tokyo, Japan) was used as a distal access catheter, through which a DeFrictor Nano microcatheter (Medico’s Hirata Co., Ltd., Tokyo, Japan) was advanced as distally as possible into the retrocorporeal artery over a Chikai X010 microguidewire (Asahi Intecc Co., Ltd., Nagoya, Aichi, Japan). Transarterial embolization was performed using Onyx 18. A total of 3.16 mL of Onyx was injected, reaching just proximal to the origin of the radiculomedullary vein (Figure 2C). Post-procedural angiography of the left L2 segmental artery confirmed the disappearance of reflux into the RMedV. Considering the high radiation dose administered, the initial procedure was concluded at this point (Figure 2D).

**Figure 3:**
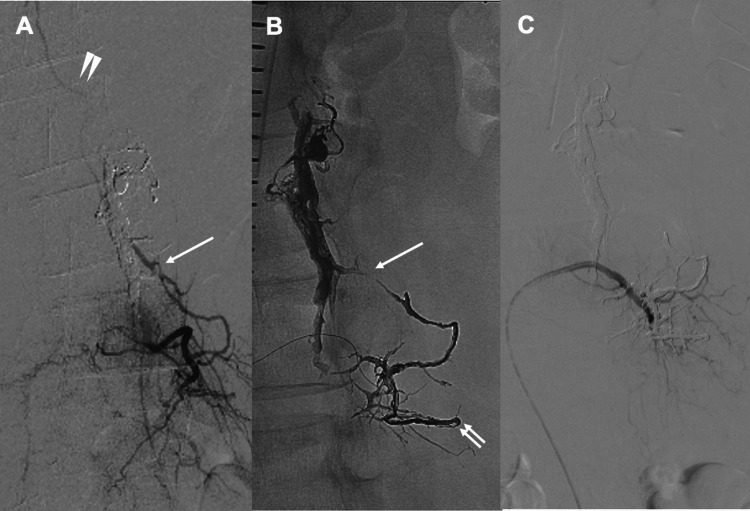
Intraoperative imaging during the second treatment (TAE) A) Selective angiography of the left L3 segmental artery demonstrated that the dorsal branch reversed its course at the tip of the transverse process and ascended, functioning as a feeder entering the origin of the same RMedV. Newly developed retrograde drainage into the identical RMedV (white arrow) was also observed. B) The microcatheter was advanced distally to the tip of the transverse process (white double arrow), and Onyx18 was used for embolization. C) Selective angiography of the left L3 segmental artery showing the disappearance of reflux into the RMedV. RMedV: Radiculomedullary vein; TAE: Transcatheter arterial embolization

Postoperatively, paraplegia showed only slight improvement, and an MRI performed four weeks later demonstrated no reduction in spinal cord edema. Suspecting recanalization, an additional treatment was performed approximately six weeks after the initial procedure. During the second session, selective angiography of the left L3 segmental artery, one vertebral level caudal to the previous lesion, revealed that the dorsal branch changed direction at the tip of the transverse process and ascended, functioning as a feeder entering the origin of the same RMedV as the previous lesion and draining retrogradely into the RMedV (Figure 3A). Using a 4-Fr NUC1 catheter, the DeFrictor Nano microcatheter was advanced over a Chikai X010 microguidewire to the distal tip of the transverse process, followed by TAE with Onyx 18 (Figure 3B). Angiography from the left L3 segmental artery demonstrated the disappearance of reflux into the RMedV (Figure 3C).

**Figure 4:**
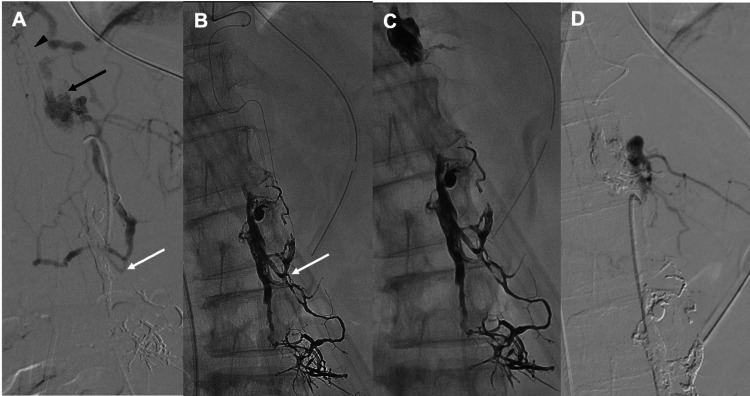
Intraoperative imaging during TVE treatment A) Selective angiography of the left Th12 segmental artery performed after the second TAE demonstrated arterial inflow into the dilated epidural venous plexus at the Th12 level (black arrow), which drained through the Th12–L1 intervertebral vein into the hemiazygos vein. The hemiazygos vein descended and connected via the L2–L3 intervertebral vein to the previously Onyx-embolized epidural venous plexus and the origin of the RMedV (white arrow) with retrograde reflux. B) The microcatheter was advanced into the RMedV (white arrow) via the azygos vein, hemiazygos vein, Th11–Th12 intervertebral vein, internal vertebral venous plexus, Th12–L1 intervertebral vein, and hemiazygos vein. C) TVE using Onyx 34 confirmed occlusion at the RMedV origin. Since the venous plexus at the Th12 level was found to be continuous with the RMedV, the microcatheter was left in the Th12 venous plexus during withdrawal, and additional Onyx 34 was injected to achieve cast formation. D) Postoperative angiography showing reflux disappearance into the RMedV. RMedV: Radiculomedullary vein; TVE: Transvenous embolization; TAE: Transcatheter arterial embolization

However, subsequent imaging revealed a newly identified route from the left Th12 segmental artery to the same RMedV that had not been detected during prior endovascular evaluations (Figure 4A). Selective angiography of the left Th12 segmental artery demonstrated that a retrocorporeal artery shunted into the dilated epidural venous plexus at the Th12 level and drained via the Th12-L1 intervertebral veins into the azygos vein. The azygos vein descended and connected through the L2-L3 intervertebral veins to the previously Onyx-cast epidural venous plexus and the origin of the RMedV, resulting in retrograde flow into the RMedV. Because the anterior spinal artery was also visualized, TAE was considered to pose a risk of spinal cord infarction; therefore, TVE was selected.

An additional right femoral vein puncture was performed, and an 8-Fr short sheath was inserted. A 6-Fr DD6 catheter (Medikit Co., Ltd., Tokyo, Japan) was advanced over an 8-Fr Roadmaster guiding catheter (Goodman Co., Ltd., Aichi, Japan) into the superior vena cava and subsequently into the azygos venous system. Using a Tactics Plus catheter (Technocrat Corporation, Aichi, Japan) as a distal access catheter, the DeFrictor Nano microcatheter was advanced downward through the azygos vein, passed through the intervertebral veins at Th11 and Th12 into the internal vertebral venous plexus, reentered the hemiazygos vein via the Th12-L1 intervertebral vein, and was finally guided into the refluxing RMedV through the L2-L3 intervertebral vein (Figure 4B). Because the microcatheter could be advanced directly into the refluxed RMedV itself, a high-viscosity embolic agent, Onyx 34, was selected, and TVE with Onyx 34 successfully occluded the RMedV. To prevent recurrence, the venous pouch at the left Th12 level was also occluded with Onyx 34 (Figure 4C). Post-procedural angiography confirmed the complete disappearance of reflux into the RMedV (Figure 4D).

**Figure 5:**
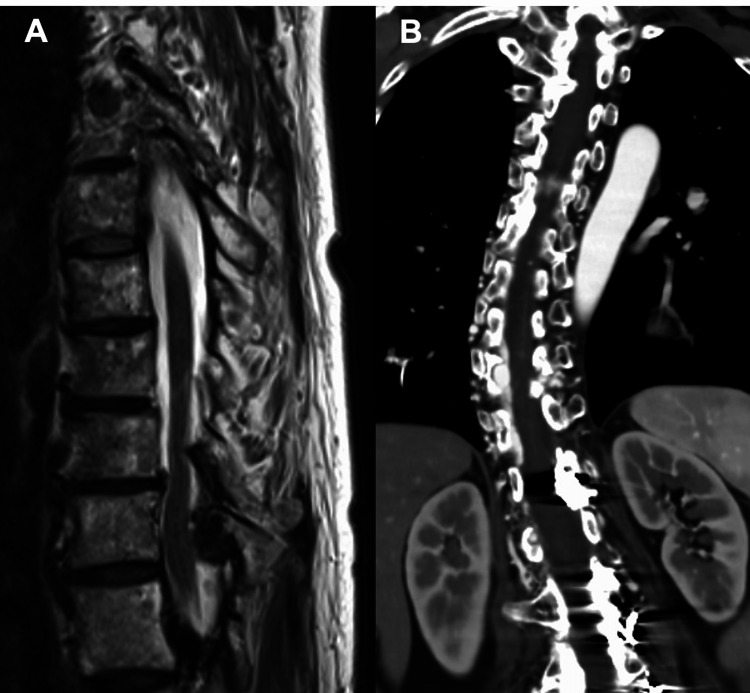
Thoracolumbar spinal MRI and coronary CTA findings after treatment A) Sagittal T2-weighted MRI showing improvement of spinal cord edema and flow voids. B) On arterial phase CTA, the previously observed spinal veins disappeared.

Follow-up imaging demonstrated radiological improvement, and the patient’s neurological condition gradually improved, allowing ambulation with a cane (Figure 5A, 5B). The patient maintained a favorable clinical status at the one-year follow-up after the final treatment.

## Discussion

This case involved an SEDAVF with an extremely complex vascular architecture, characterized by multiple segmented and discontinuous epidural venous plexuses spanning seven vertebral levels. Such extensive and fragmented involvement made anatomical interpretation highly challenging and rendered complete obliteration of all shunts impractical. The four epidural venous plexuses observed in this case appeared discontinuous on imaging and collectively extended across seven vertebral bodies, a pattern that markedly differs from the typically reported single and relatively localized epidural venous pouch morphology [2]. Although previous studies have described SEDAVFs with complex venous drainage pathways [5], lesions spanning multiple vertebral levels [9], large venous pouches eroding vertebral bodies (the so-called “osseous type”) [10], or cases in which an underlying congenital disorder such as spinal arteriovenous metameric syndrome (SAMS) was considered in the presence of multilevel shunts, the present case is distinct [11]. In our case, no physical findings suggestive of a congenital vascular disorder were identified, making a congenital etiology unlikely. To the best of our knowledge, no previous reports have described a SEDAVF simultaneously exhibiting multiple extensive and discontinuous epidural venous plexuses spanning seven vertebral levels, as observed in the present case.

Based on venous drainage patterns, SEDAVFs are classified into lesions draining purely into the epidural or paravertebral veins and often remaining asymptomatic (Type B), those causing myelopathy due to reflux into the intradural venous system, primarily the perimedullary veins (Type A), and lesions exhibiting a combination of both patterns [6]. Two principal mechanisms underlie symptom development: congestive myelopathy caused by intradural venous reflux and compressive radiculopathy or myelopathy resulting from enlarged epidural venous structures [2]. The former accounts for approximately 90% of symptomatic cases and is typically associated with rapid neurological deterioration [3]. Physiologically, reflux from the epidural to the intradural venous system is normally prevented by functional narrowing of the radicular veins as they traverse the dura mater. However, because the epidural venous plexus lacks valves, high-flow shunts can result in arterialization of epidural veins; subsequent thrombosis or occlusion may disrupt this protective mechanism, allowing intradural reflux and venous hypertension that can acutely induce severe congestive myelopathy [12]. In the present case, the rapid progression of neurological symptoms strongly suggested intradural venous reflux as the primary mechanism underlying the clinical presentation.

The conventional therapeutic goal in SEDAVF is complete obliteration of the arteriovenous shunt, including interruption of both the arterialized epidural venous pouch and the intradural radicular draining veins [6]. In most reported cases, embolization targeting all shunt points and associated dilated venous plexuses has been performed. However, in the present case, embolization of all dilated venous plexuses was neither feasible nor considered necessary because of the extremely extensive distribution of the lesions and the determination that intradural venous reflux was the sole contributor to the patient’s symptoms. By focusing treatment on the symptom-causing lesion rather than attempting eradication of the entire disease, long-term symptomatic improvement was achieved despite incomplete elimination of all shunt components. Residual SEDAVFs without reflux into the perimedullary veins are generally considered clinically benign in the absence of compressive symptoms and have been reported to undergo spontaneous thrombosis or closure without recurrence during long-term follow-up [7,13]. Given the sustained clinical improvement in our patient, careful observation was deemed appropriate.

Endovascular embolization is the most commonly employed treatment modality for SEDAVF, reportedly used in approximately two-thirds of cases [3,14], with complete occlusion rates ranging from 73.3% to 94.4% and symptomatic improvement observed in 62.5%-91% of patients [13,15]. Nevertheless, endovascular treatment has limitations, including relatively high recurrence rates [13], the potentially large volume of liquid embolic material required for extensive epidural venous pouches, and the risk of spinal cord infarction during TAE when the shunt shares arterial supply with the anterior spinal artery [15]. In the present case, although Onyx penetration into the radiculopial vein origin and subsequent disappearance of the radiculopial vein were confirmed after the initial treatment, the embolic agent failed to adequately penetrate the radiculomedullary vein, necessitating retreatment. Similar phenomena have been reported, including the emergence of new feeders after embolization [16]and recurrence related to insufficient penetration of embolic material into the fistulous point or draining veins [17]. Furthermore, the need for embolization from the same segmental artery supplying the anterior spinal artery during the second session prompted a strategic shift from TAE to TVE.

In TVE, when the internal vertebral venous plexus is thrombosed and segmented, the radiculomedullary vein can be accessed by navigating through the azygos vein, hemiazygos vein, and internal vertebral venous plexus via the intervertebral veins [18]. Careful preoperative imaging evaluation, including not only angiography but also three-dimensional CT angiography, is essential for identifying a safe and feasible venous access route. In the present case, embolization was deliberately limited to the region responsible for reflux into the radiculomedullary vein, achieving effective clinical improvement while minimizing radiation exposure and reducing the volume of Onyx required. In SEDAVFs with extensive and complex vascular architecture involving multiple shunts, as demonstrated here, complete anatomical understanding and total shunt obliteration may be unrealistic. Nevertheless, a cause-oriented treatment strategy focusing on the symptom-producing lesion may provide durable symptomatic improvement and represents a pragmatic and effective therapeutic option.

## Conclusions

In SEDAVF cases with multiple expanded venous plexuses spanning several vertebral levels, identifying the responsible lesion is challenging due to the anatomical complexity, making detailed preoperative imaging analysis and accurate determination of the symptomatic source essential. Although transvenous embolization offers a high likelihood of achieving a complete cure, the initial stage of treatment often involves numerous feeders and difficulties in selecting an appropriate venous access route. Therefore, performing transarterial embolization first and subsequently adding transvenous embolization when the result is insufficient represents a reasonable therapeutic option.
